# The importance of an idiographic approach for the severe chronic disorders—the case of the amyotrophic lateral sclerosis patient

**DOI:** 10.3389/fpsyg.2012.00509

**Published:** 2012-11-19

**Authors:** F. Pagnini, C. J. Gibbons, Gianluca Castelnuovo

**Affiliations:** ^1^Department of Psychology, Catholic University of MilanMilan, Italy; ^2^Niguarda Ca' Granda HospitalMilan, Italy; ^3^The University of ManchesterManchester, UK; ^4^Psychology Research Laboratory, Ospedale San GiuseppeVerbania, Italy

The discussion about nomothetic and idiographic approaches is one of the main philosophical debates in the field of psychological sciences (Schafer, 1999; Salvatore and Valsiner, 2010).

The term “nomothetic” comes from the Greek word “nomos” that means “usage, custom, law” and in psychology is referred to the objective classification under similar conditions, to in establish generalizations, such as diagnoses. The term “idiographic” derives from the Greek word “idios”, meaning “pertaining to self; one's own, private or separate” and is referred to the aspects of subjective experience that makes each person unique.

In the clinical psychology, the debate deals about the classification of personality and other taxonomies, as well as the use of diagnosis. At the intervention level, the discussion is about the use of individualistic treatment techniques or treatment derived from research where the focus is a group of people with a similar condition, where (individual) efficacy is tested with aggregated statistics. For example, evidence-based research indicates that the cognitive-behavioral therapy has a positive impact toward the anxiety disorders (Olatunji et al., 2010).

As researchers, we look for regularities and repetitive phenomena. However, when the study object is the human being, with all his complexity, we cannot forget that every person is unique. That is even more important in the clinical practice, where clinicians do not have to take care of an “average score,” but they must assist an individual, with his clinical peculiarities, thoughts, emotions and relationships.

When dealing with severe and chronic illnesses, such as Amyotrophic Lateral Sclerosis (ALS), this debate is very important and sometimes is at risk of being underestimated. The human tendency toward generalization, together with a superficial approach to research findings, may lead us to stereotypical beliefs about a person living with severe disease. In the case of ALS, an incurable terminal illness with a potentially steep decline in physical function and erosion of independence, it could easily be assumed that “if a person has ALS, than he/she must be depressed and have a low quality of life”(Rabkin et al., 2009).

The reaction toward a grave diagnosis such as ALS will vary greatly from person to person. Individual's characteristics will lead to a personal reaction that may include depressive features, despair, hopelessness, but there remains potential for the individual to find hope, and maintain hope in the face of such serious prognosis (McDonald et al., 1994). Therefore, even if we can find out, statistically, the average score of each psychological issue involved, no aggregate research will be able to consider the whole complexity of the individual reaction.

The practice of clinical psychology use to be guided by an idiographic approach, with an attention to research results derived from a nomothetic point of view. However, in certain cases, prejudices for a clinical situation may interfere in the process of individual knowledge. In the ALS field, considering the low prevalence of the disease, it is possible that a professional without a good deal of knowledge about such illnesses may overestimate depressive features based on assumption. A similar concept is also important for other types or clinicians, such as the physicians, in particular when they are not used to treat a particular clinical situation.

One way of better understanding a patient's world is to complete a quality of life (QoL) questionnaire, though care should be taken when selecting a questionnaire for this purpose. QoL is defined by the World Health Organisation as a “broad ranging concept affected in a complex way by the person's physical health, psychological state, level of independence, social relationships, personal beliefs, and their relationship to salient features of their environment” (WHOQOL Group, 1998). There are two different conceptualizations of QoL, known as health-related Quality of Life (HRQoL), and subjective well-being (SWB). HRQoL largely quantifies QoL based patient self-reported symptoms and ability to carry out activities of daily living. Subjective well-being, otherwise known as “Individual Quality of Life” in ALS research literature (Clarke et al., 2001), refers to a positive state of mind and satisfaction with life in general, and is not contingent on assessment of symptom severity. According to the construct of HRQoL and the manner in which it conceptualizes QoL, patients with ALS *must* have a lower QoL than the general population, with further decreases as symptom severity increases, in line with disease progression. However, past research has shown that QoL is unrelated to physical strength and the functional capacity (Goldstein et al., 2002; Pagnini, 2012). Therefore, when administering a QoL measure, clinicians must be aware of the subtle, but very important distinction between QoL and the confusingly named HRQoL. Ideally, clinicians should always administer questionnaires that have been shown to work well with the intended illness group. Even questionnaires that are apparently suitable and ostensibly avoid somatic symptoms of depression have been shown to overestimate depressive features for ALS patients (Gibbons et al., 2011).

Even if researchers may take advantages from standardized questions and replies, clinicians should also include a patient-based focus. In the assessment of the QoL, for example, there are many questionnaires that are useful to understand how a person feels, with different degrees of sensitivity. One of the most interesting instruments for the assessment of the individual needs, beliefs and emotions, is the Schedule for the Evaluation of Individual Quality of Life (SEIQoL) (Hickey et al., 1996) that asks people to indicate the five most relevant domains for their well-being, to rate them and to classify among each other. Even if the score obtained has been criticized (Felgoise et al., 2009) and has some limitations from the research point of view, the clinical process of domain selection and rating may provide interesting insights about the individual's situation and prove useful in the formulation process, coherently with a SWB approach. The use of a similar approach by clinical psychologists and physicians who work with chronic illnesses may allow assist in understand the patient's individual clinical issues, reducing prejudices and the risk of a blind application of a nomothetic approach.
